# Schisandrin B alleviates high-glucose diet-induced oxidative stress, inflammation, and tissue injury in zebrafish and is associated with alterations in intestinal microbiota

**DOI:** 10.3389/fvets.2026.1767061

**Published:** 2026-05-08

**Authors:** Miao Fu, Wei Xu, Changmin Deng, Anhuai Yang, Mingyang Xue, Xuan Xiao, Lijuan Xu

**Affiliations:** 1 Center of Health Management, Renmin Hospital of Wuhan University, Wuhan, China; 2 Department of Ophthalmology, Renmin Hospital of Wuhan University, Wuhan, China; 3 Yangtze River Fisheries Research Institute, Chinese Academy of Fishery Sciences, Wuhan, China

**Keywords:** high-glucose diet, inflammation, intestinal microbiota, oxidative stress, Schisandrin B

## Abstract

High-glucose diet (HGD) has been reported to alter gut microbiota and induce chronic inflammation and oxidative stress. In this study, we demonstrated that Schisandrin B (SchB) markedly attenuated HGD-induced physiological disturbances in zebrafish, with a focus on multiple tissues, including the liver, intestine, brain, and retina. The results showed that SchB attenuated oxidative stress, inflammatory responses, and tissue injury induced by HGD. SchB was associated with reduced levels of reactive oxygen species (ROS) and malondialdehyde (MDA) levels, as well as enhanced superoxide dismutase (SOD) activity. In addition, the mRNA expression levels of oxidative stress–related genes (Nrf2 and HO-1) and inflammatory markers (IL-1β, IL-8, TNF-*α*, and Myd88) were significantly modulated in SchB-treated groups. Intestinal microbiota analysis revealed that SchB was associated with increased microbial richness and diversity, as well as partial restoration of HGD-induced dysbiosis. Overall, these findings suggest that SchB exerts antioxidant and anti-inflammatory effects in zebrafish under high-glucose dietary conditions, and these protective effects are associated with improvements in gut microbiota structure.

## 1 Introduction

A high-glucose diet (HGD) has been strongly linked to the onset of multiple metabolic disorders, such as diabetes, obesity, and cardiovascular conditions (1). Growing evidence from animal and cell-based studies indicates that high glucose exposure can trigger persistent inflammation and oxidative stress, leading to the activation of multiple pro-inflammatory mediators, including cytokines such as interleukin-6 (IL-6) and IL-8 (2). In addition, long-term hyperglycemia enhances glucose oxidation and promotes the nonenzymatic glycation of proteins, after which the oxidized glycated proteins undergo further degradation, ultimately causing an overproduction of free radicals (3). In the context of oxidative and antioxidant reactions, intracellular ROS, MDA, and antioxidant enzymes like SOD are crucial regulators of the pro-inflammatory signaling pathways triggered by high-glucose exposure (4).

Free radicals derived from oxygen and nitrogen are essential for aerobic organisms, and ROS participate in fundamental biochemical processes that help maintain redox homeostasis within tissues and cells. Physiological health is of paramount importance for organisms. Nevertheless, excessive oxidative stress can damage lipids, proteins, and DNA, ultimately impairing organismal health (5). Oxidative stress occurs when the equilibrium between pro-oxidant processes and antioxidant defenses becomes disturbed, leading to the degradation of intracellular macromolecules such as carbohydrates, lipids, proteins, and nucleic acids. It subsequently leads to organ and tissue damage and functional impairment. This, in turn, leads to the further accumulation of oxidation products, exacerbating the damage and creating a vicious cycle (6). Nuclear factor erythroid 2–related factor 2 (Nrf2) is a redox-responsive transcription factor that is broadly expressed across different cell types (7, 8). The Nrf2 pathway governs the expression of more than 600 genes, among which over 200 codes for proteins implicated in inflammation, tumor development, neurodegenerative conditions, aging, cardiovascular disorders, and other major diseases (9). Through regulation of multiple downstream targets, Nrf2 mediates diverse protective effects such as anti-inflammatory responses, attenuation of calcium overload, and antioxidative stress activity. Heme oxygenase-1 (HO-1) is a vital and extensively studied antioxidant gene regulated by Nrf2. It combines with biliverdin reductase to release bilirubin, which stands as one of the most potent endogenous antioxidants, effectively neutralizing ROS and reactive nitrogen species (RNS) (10). Antioxidant defense systems help counteract the pathological damage caused by hyperglycemia-induced oxidative stress and can slow the progression of insulin resistance (11).

The gut microbial community is strongly linked to the onset and progression of multiple metabolic disorders due to its essential roles in host metabolism and immune regulation (12). High sugar intake is well known to alter gut microbiota composition and to promote obesity and metabolic disorders (13). Dietary modulation of the gut microbiota can elevate circulating levels of bacterial lipopolysaccharide (LPS), which has been associated with the development of insulin resistance and disturbances in glucose tolerance. In addition, microbially derived LPS is closely linked to inflammatory responses and the progression of metabolic disorders (14).

In traditional Chinese medicine, Schisandrin (Sch) refers to a group of bioactive lignans—primarily Schisandrin A, B, and C—recognized for their broad preventive and therapeutic properties (15, 16). Recent pharmacological studies suggest that SchB exerts multiple biological effects, including hepatoprotection, antioxidative activity, anti-aging effects, antitumor properties, and anxiolytic actions (17). Experimental evidence further indicates that SchB can increase cellular SOD activity, inhibit lipid peroxidation, and reduce lactate dehydrogenase and MDA levels, thereby lowering ROS accumulation, directly scavenging free radicals, and functioning as a potent antioxidant (18, 19). In fact, SchB exhibited a significantly more potent scavenging effect on oxygen free radicals, particularly hydroxyl free radicals, compared to vitamin C at equivalent concentrations (20).

Given that a high-glucose diet disrupts redox homeostasis, activates inflammatory signaling pathways, and induces gut microbiota dysbiosis, developing effective strategies to counteract these pathological processes is of great importance. SchB has been shown to enhance antioxidant enzyme activities, suppress lipid peroxidation, and modulate inflammatory mediators, highlighting its potential as a multifunctional metabolic regulator. However, whether SchB can concurrently mitigate HGD-induced oxidative stress, attenuate inflammatory responses, and restore gut microbiota composition has not been systematically examined. Therefore, this study employed an HGD-induced zebrafish model to comprehensively evaluate the protective effects of SchB on the liver, intestine, brain, and retina, as well as its effects on pro-inflammatory cytokine expression and associated changes in gut microbial communities. These findings are expected to contribute to a better understanding of the potential of SchB as a nutritional intervention for preventing or alleviating glucose-induced metabolic disturbances.

## 2 Materials and methods

### 2.1 Fish

Adult wild-type zebrafish (*Danio rerio*) (4 months of age, 0.40 ± 0.05 g of body weight) were sourced from the China Zebrafish Resource Center (CZRC) in Wuhan, China. The fish were maintained in a Benchtop Aquatic System provided by Jinshui Marine Biological Equipment Co. in Wuhan, China, under a 12 h light/12 h dark cycle at a temperature of 28 ± 1 °C for a minimum of 14 days before commencing the study.

### 2.2 Diet preparation and sampling

SchB (purity > 98%) and D-glucose were purchased from Sigma-Aldrich (St. Louis, MO). SchB was dissolved in DMSO and then directly mixed into the basal feed at the designated concentration to prepare the SchB-supplemented diets. The same volume of DMSO was added to the control and HGD feeds to exclude potential solvent effects. For the *in vivo* toxicity assessment, zebrafish were exposed to various doses of SchB (10, 20, 30, 40, 50, and 100 mg/kg) over 28 days, with 30 fish per dose. The survival rate, behavioral changes (e.g., swimming activity, feeding behavior), and general health status were monitored daily. No significant mortality or abnormal behavioral changes were observed at doses up to 30 mg/kg. Based on these results, 30 mg/kg was selected as the experimental dose for subsequent feeding trials.

A total of 360 zebrafish were randomly allocated into four experimental groups: Group 1 (control diet, Control), Group 2 (high-glucose diet, HGD), Group 3 (control diet supplemented with 30 mg/kg Schisandrin B, SchB), and Group 4 (high-glucose diet supplemented with 30 mg/kg Schisandrin B, HGD + SchB). Each group consisted of three biological replicates, with 30 fish per replicate.

The control diet was formulated without additional glucose. The high-glucose diet (HGD) was prepared by supplementing the basal diet with 15% glucose (w/w). This level was selected with reference to previous studies in fish nutrition, where high-carbohydrate diets typically contain approximately 45% total carbohydrate (21). Considering that the basal diet used in this study contained approximately 30% carbohydrate, additional glucose was supplemented to elevate the carbohydrate level and induce metabolic stress.

On the 28th day of the feeding period, four fish were randomly sampled from each tank, anesthetized using 50 mg/L MS222 (Sigma Aldrich, St. Louis, MI, USA). For biochemical assays, gene expression analysis, and histological examination, tissues (liver, intestine, brain, and retina) were collected from individual fish, with four biological samples per replicate (*n* = 4). For microbiota analysis, intestinal contents from the four fish within each replicate were pooled to obtain one composite sample, resulting in three biological replicates per group (*n* = 3). All samples were immediately collected, snap-frozen in liquid nitrogen, and stored at −80 °C until further analysis.

### 2.3 Tissue antioxidant index

Levels of reactive oxygen species (ROS), malondialdehyde (MDA), and superoxide dismutase (SOD) activity in different tissues were measured using commercial assay kits (Jiancheng, Nanjing, China) according to the manufacturer’s instructions.

The retina was selected for antioxidant analysis due to its high sensitivity to oxidative stress and its suitability as an indicator of systemic oxidative damage.

### 2.4 Real-time quantitative PCR

Total RNA was extracted from tissues using TRIzol reagent (Thermo Fisher Scientific, Waltham, MA, USA) according to the manufacturer’s instructions. RNA concentration and purity were determined spectrophotometrically. cDNA was synthesized using a reverse transcription kit (Vazyme, Nanjing, China). Quantitative RT-PCR was performed using SYBR Green chemistry on real-time PCR system. Gene-specific primers are listed in Table 1. Relative gene expression levels (including IL-1*β*, IL-8, TNF-*α*, Myd88, Nrf2, and HO-1) were calculated using the 2^^−ΔΔCt^ method, with β-actin as the internal reference gene.

**Table 1:** The primers.

Gene name	Accession no.	Primer sequences (5′ → 3′)
IL-1β	NM_212844.2	F: AAAGTGCGCTTCAGCATGTC
		R: GCTGGTCGTATCCGTTTGGA
IL-8	NM_001327985.1	F: GCCTTCATGCTTCTGATCTGC
		R: AATCACCCACGTCTCGGTAGGA
TNF-α	AB183467	F: CACAAAGGCTGCCATTCACT
		R: GATTGATGGTGTGGCTCAGGT
Myd88	NM_212814.2	F: GTGATGCCTGTGATTTTCAGACTAA
		R: CGGCCTCTTCATGGATTTGT
Nrf2	NC_007120.7	F: AACGAGTTCTCCCTTCAGCA
		R: ATTTTGTCGCCGATTTTGTC
HO-1	NC_007114.7	F: GGAAGAGCTGGACAGAAACG
		R: CGAAGAAGTGCTCCAAGTCC

### 2.5 Histological analysis

Intestinal and liver tissues were fixed in 4% paraformaldehyde for 24 h, dehydrated through a graded ethanol series (50–95%), and embedded in paraffin. Paraffin blocks were sectioned at a thickness of 5 μm and stained with hematoxylin and eosin (HE). The morphology of the intestinal tissue and hepatocytes was observed, and images were captured using a BX53 microscope (Olympus, Tokyo, Japan).

### 2.6 Intestinal microbiota analysis

Microbial genomic DNA from intestinal bacteria was extracted using a bacterial DNA extraction kit (Omega, Norcross, USA). The V3–V4 region of the bacterial 16S rDNA was amplified in 50 μL PCR reactions with barcoded primers 338F and 806R. The amplification program consisted of an initial denaturation at 95 °C for 1 min, followed by 30 cycles of 95 °C for 30 s, 55 °C for 30 s, and 72 °C for 45 s, with a final extension at 72 °C for 10 min. PCR products were separated on 2% agarose gels and purified using a DNA gel extraction kit (Omega, Norcross, USA). The resulting amplicons were then subjected to high-throughput sequencing on an Illumina MiSeq platform.

Sequence reads were subjected to quality control and assembly using the Mothur software package (version v.1.30) (22). The processed reads were subsequently clustered into operational taxonomic units (OTUs) based on a 97% sequence similarity cutoff (23). Alpha diversity metrics, including the abundance-based coverage estimator (ACE), Chao1, Shannon, and Simpson indices, were calculated with Mothur (24). Beta diversity was assessed based on Bray–Curtis distances calculated from genus-level relative abundance data and visualized using principal coordinate analysis (PCoA). For functional phenotype prediction, OTU tables were generated using a closed-reference approach, and the potential pathogenicity of predicted microbial phenotypes was evaluated using BugBase (25).

The sequencing data have been deposited in the Genome Sequence Archive (GSA) of the China National Center for Bioinformation (CNCB-NGDC) under accession number PRJCA062488.

### 2.7 Statistical analyses

Statistical analyses were conducted in SPSS 26.0 (SPSS Inc., Chicago, IL, USA). Data are presented as mean ± standard deviation or median with interquartile range, as appropriate. Differences among groups were assessed using Student’s t-test or one-way analysis of variance (ANOVA) followed by Tukey’s multiple comparison test, as appropriate. A *p*-value < 0.05 was considered statistically significant.

## 3 Results

### 3.1 Oxidative and antioxidant indexes

**Figure 1:**
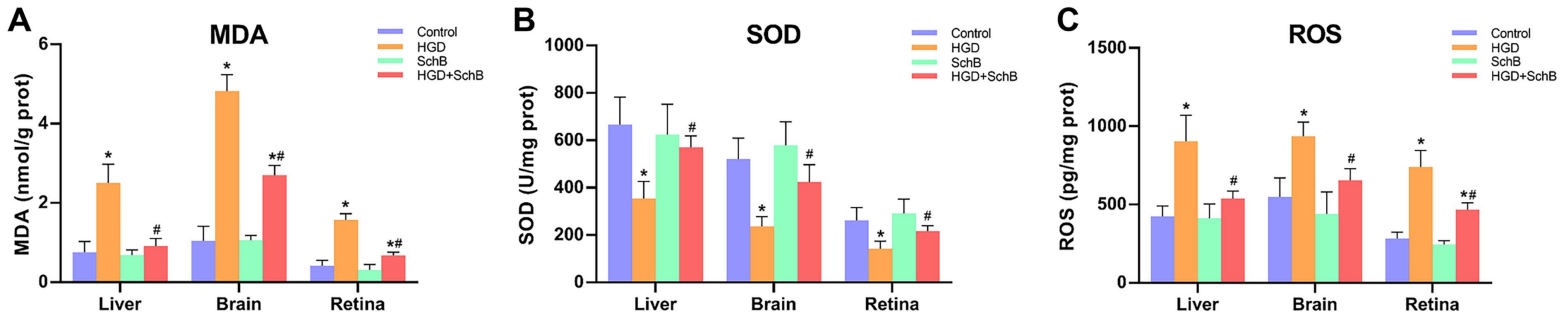
Oxidative and antioxidant indices in zebrafish tissues. **(A)** MDA levels in liver, brain, and retina. **(B)** SOD activity in the liver, brain, and retina. **(C)** ROS levels in the liver, brain, and retina. Data are presented as mean ± standard deviation. ^*^p < 0.05 vs. Control group; ^#^*p* < 0.05 vs. HGD group.

Figure 1 shows that the HGD group exhibited significantly elevated ROS and MDA levels, accompanied by markedly reduced SOD activity in the retina, brain, and liver compared with the Control group. Following SchB supplementation (HGD + SchB group), MDA levels decreased by approximately 64, 44, and 57% in the liver, brain, and retina, respectively, while ROS levels were reduced by around 40, 30, and 37% in these tissues. Concurrently, SOD activity increased by about 61, 79, and 52% in the retina, brain, and liver, respectively.

### 3.2 SchB supplementation mitigates HGD-induced activation of the Nrf2/HO-1 pathway

**Figure 2:**
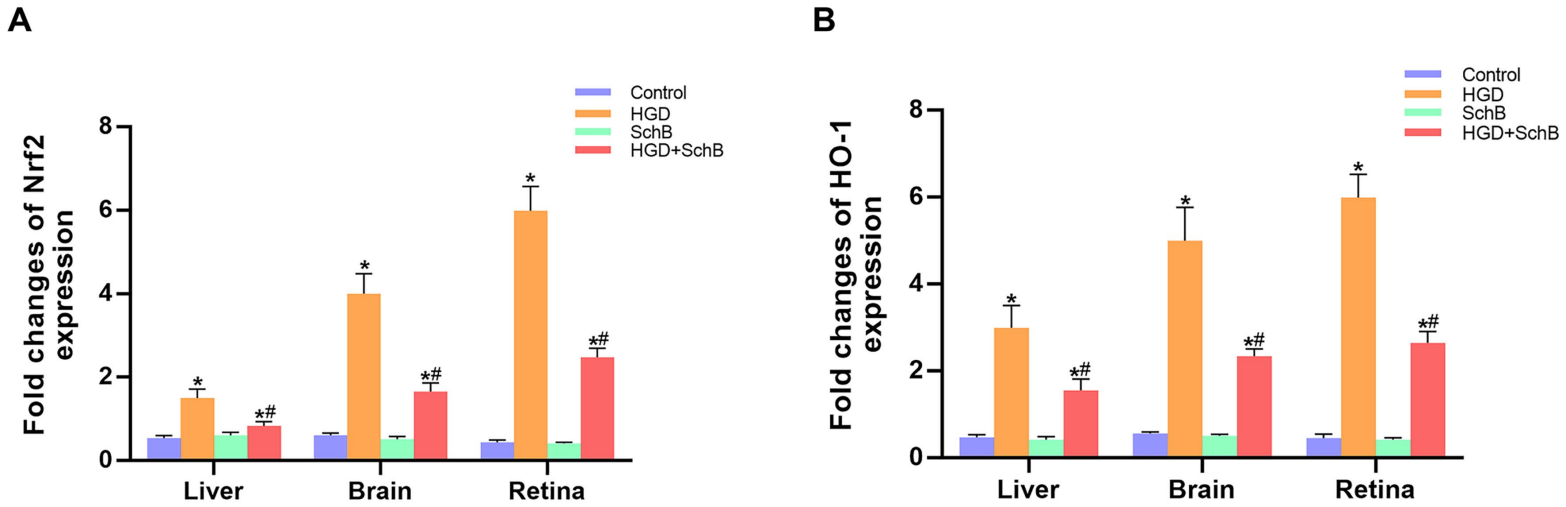
Effects of SchB on the mRNA expression of oxidative stress–related genes in zebrafish. **(A)** The fold changes of Nrf2 expression in liver, brain, and retina. **(B)** The fold changes of HO-1 expression in liver, brain, and retina. Results are presented as mean ± standard deviation. ^*^p < 0.05 vs. control group; ^#^*p* < 0.05 vs. cHGD group.

Figure 2 shows that HGD markedly upregulated the expression of Nrf2 and HO-1 in the liver, brain, and retina of zebrafish compared with the Control group. SchB supplementation effectively attenuated this oxidative stress response. In the HGD + SchB group, Nrf2 expression was significantly decreased across all examined tissues relative to the HGD group, accompanied by a corresponding reduction in HO-1 expression.

### 3.3 Anti-inflammatory effect of SchB

**Figure 3:**
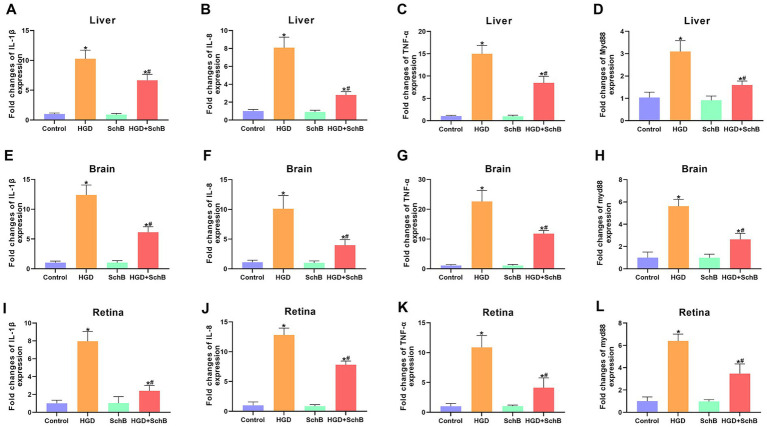
Effects of SchB on inflammation-related gene expression in zebrafish tissues. **(A–L)** Represent the expression levels of inflammation-related genes (IL-1β, IL-8, TNF-*α*, and Myd88) in different organs of zebrafish subjected to various treatments. Data are expressed as mean ± SD. ^*^p < 0.05 vs. ontrol group; ^#^*p* < 0.05 vs. cHGD group.

Exposure to a high-glucose diet (HGD) markedly elevated the expression levels of IL-1β, IL-8, TNF-*α*, and Myd88 in the liver, brain, and retina compared with the Control group. In contrast, SchB supplementation effectively mitigated this inflammatory activation. In the HGD + SchB group, the expression of IL-1β, IL-8, TNF-α, and Myd88 was significantly reduced across all three tissues, indicating a pronounced anti-inflammatory effect of SchB (Figure 3).

### 3.4 Liver and intestinal morphological observations

**Figure 4:**
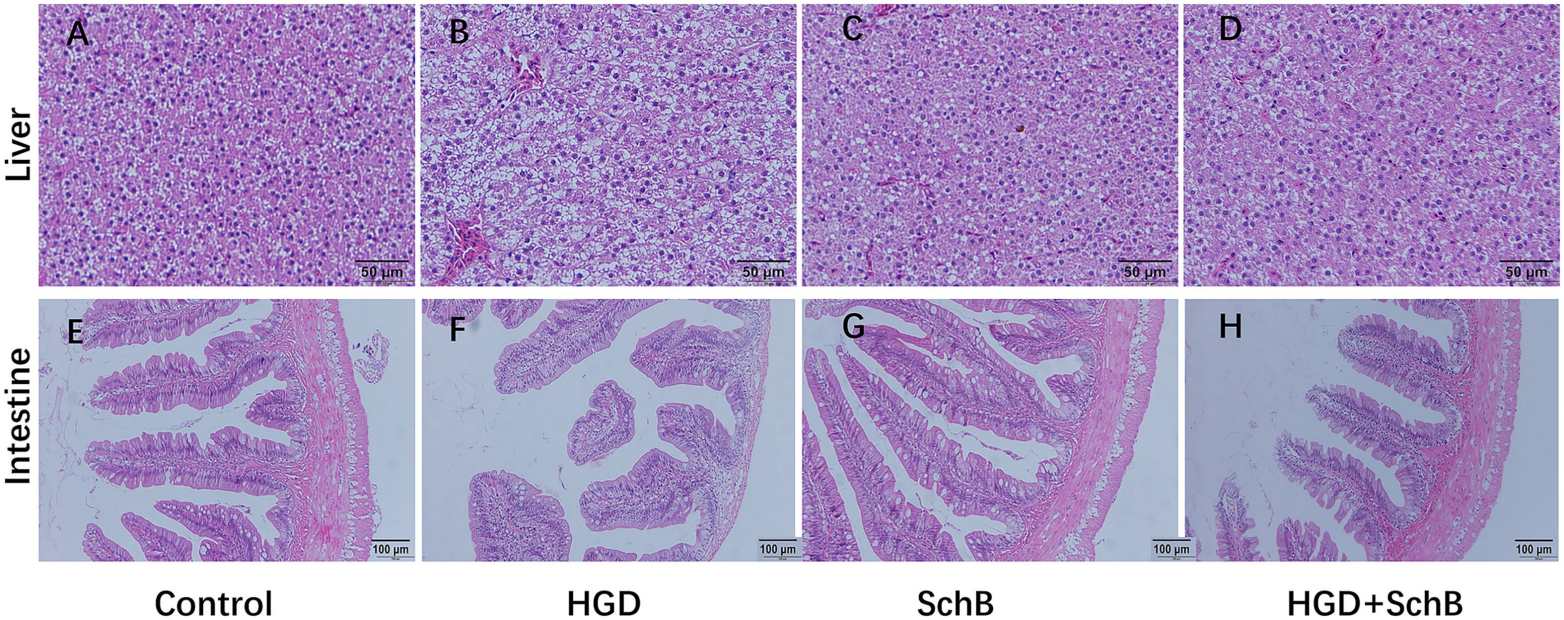
Histological analysis of the intestine and liver in zebrafish. **(A, E)** Control group; **(B, F)** HGD groups; **(C, G)** SchB groups; **(D, H)** HGD + SchB groups.

Histological findings for the liver and intestine are shown in Figure 4. Compared with the control group (Figure 4A), the HGD group exhibited pronounced vacuolar degeneration and marked inflammatory cell infiltration in the liver (Figure 4B). In the SchB alone group, no obvious liver damage was observed (Figure 4C). Administration of SchB alleviated HGD‑induced hepatic vacuolation and inflammation (Figure 4D). Regarding the intestine, the control group showed normal morphology (Figure 4E). Compared with the control, the HGD group displayed impaired intestinal architecture, characterized by thinner intestinal walls and shorter intestinal villi (Figure 4F). The SchB alone group did not show significant intestinal injury (Figure 4G). Following SchB supplementation, intestinal villus height and wall thickness increased significantly relative to the HGD group (Figure 4H).

### 3.5 Effects of SchB on alpha diversity of intestinal microbiota

**Figure 5:**
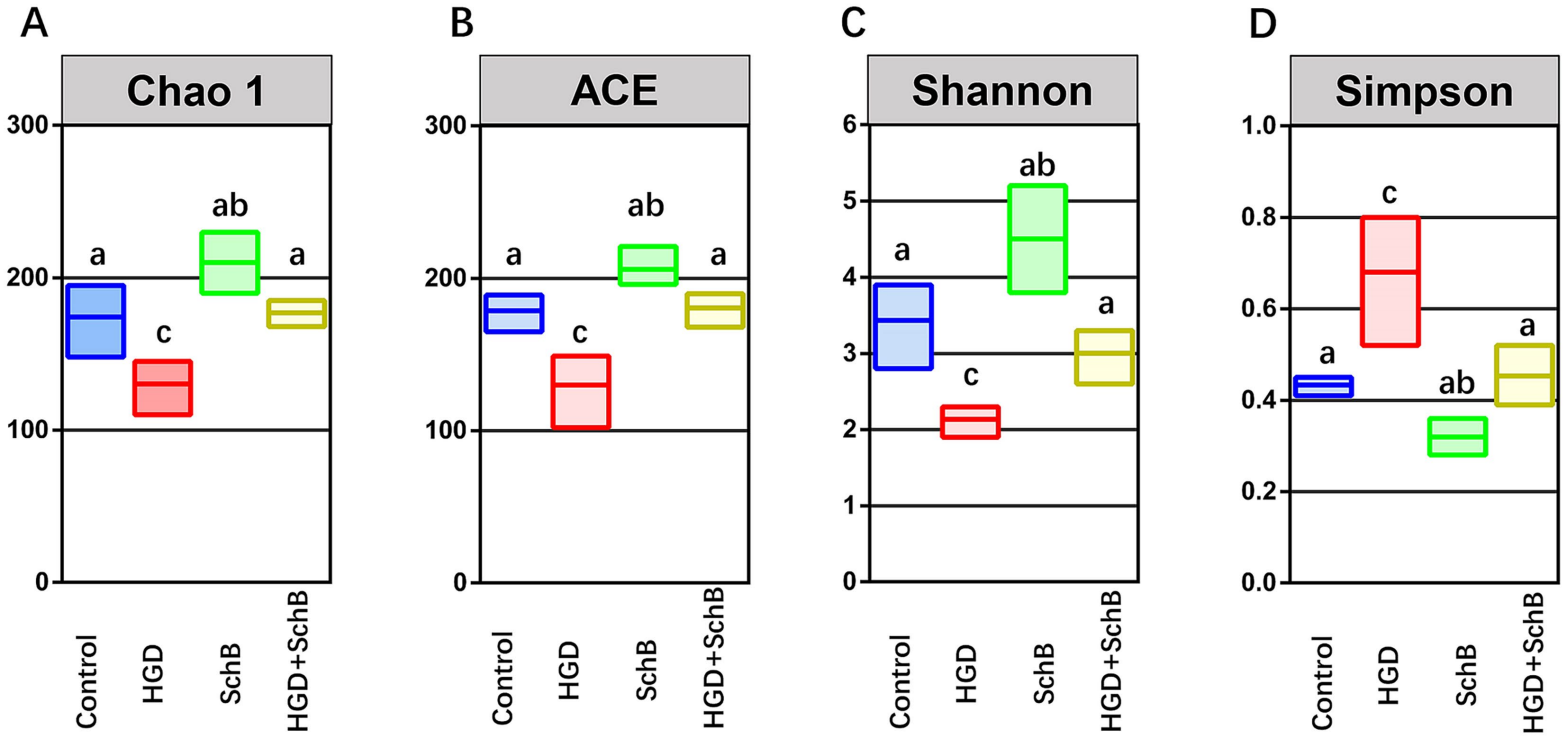
Richness and diversity of the intestinal microbiota in zebrafish from different experimental groups. **(A)** Chao1 index; **(B)** ACE index; **(C)** Shannon index; **(D)** Simpson index (dominance form). Data are presented as median with interquartile range (*n* = 3). Differences among groups were analyzed using one-way ANOVA followed by Tukey’s multiple comparison test. Different letters above boxes indicate significant differences (*p* < 0.05).

To evaluate changes in microbial richness, we applied both the ACE and Chao1 estimators to the intestinal microbial communities. The calculated Chao1 values fell within a range of approximately 110–230, whereas ACE values varied from 102 to 221 (Figure 5). One-way ANOVA revealed significant differences in Chao1 and ACE indices among groups (*p* < 0.05). Compared with the control group, microbial richness was significantly reduced in the HGD group and partially restored following SchB supplementation.

Overall community diversity was further assessed using the Shannon and Simpson indices. The Shannon metric spanned from roughly 1.9–5.2, while the Simpson index (dominance form) ranged between 0.28 and 0.80 across samples. One-way ANOVA revealed significant differences in Simpson and Shannon indices among groups (*p* < 0.05). Zebrafish receiving SchB exhibited increased Shannon diversity and decreased Simpson index values compared with the HGD group, indicating improved community evenness and reduced dominance of specific taxa (Figure 5). In contrast, prolonged exposure to a high-glucose diet significantly suppressed Shannon diversity and increased Simpson index values, reflecting reduced ecological complexity and microbial imbalance. Collectively, these results indicate that SchB alleviates HGD-induced impairments in gut microbial diversity and contributes to the restoration of a more balanced intestinal microbiota.

### 3.6 Microbial community composition

**Figure 6:**
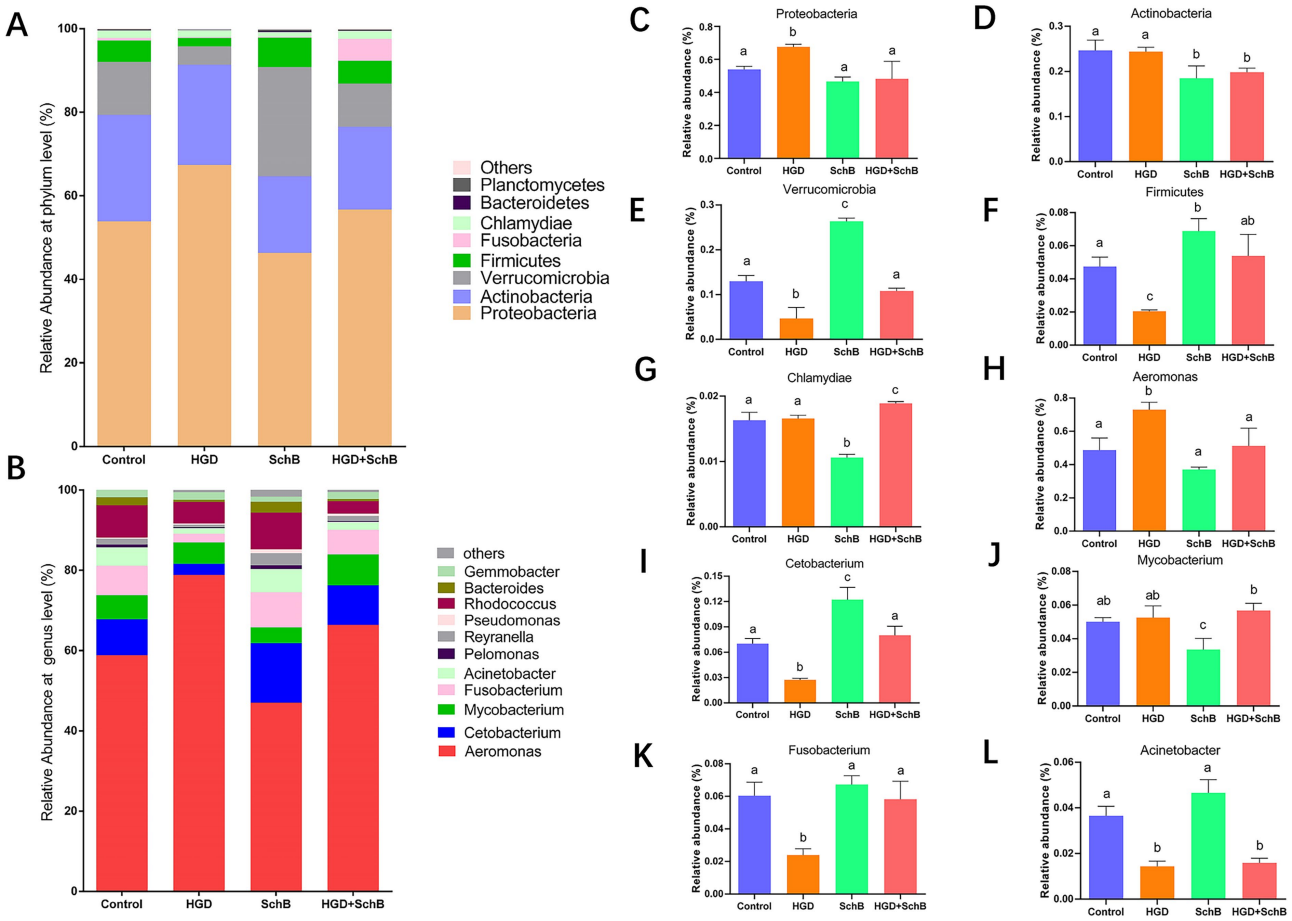
The structure and composition of the intestinal microbiota of zebrafish in different groups at phylum level **(A)** and at the genus level **(B)**. **(C–G)** Relative abundance of representative differential bacterial phyla among groups. **(H–L)** Relative abundance of representative differential bacterial genera among groups. Data are presented as mean ± SD. Different letters above bars indicate significant differences among groups (*p* < 0.05).

OTUs derived from intestinal microbial communities were analyzed for each experimental group at both the phylum and genus levels. At the phylum level, four dominant lineages—*Proteobacteria*, *Actinobacteria*, *Verrucomicrobia*, and *Firmicutes*—accounted for the majority of sequences across all samples (Figure 6A). However, the relative abundance of these major taxa differed markedly among dietary treatments. High-glucose feeding was associated with a pronounced enrichment of Proteobacteria, a phylum commonly linked to intestinal dysbiosis and inflammatory conditions. Notably, this enrichment was significantly attenuated in zebrafish receiving SchB supplementation (Figures 6C–G). In contrast, *Verrucomicrobia* and *Firmicutes* exhibited reduced relative abundance under HGD exposure but showed partial recovery following SchB treatment.

At the genus level, *Aeromonas*, *Cetobacterium*, *Mycobacterium*, *Fusobacterium*, and *Acinetobacter* constituted the predominant taxa across all experimental groups (Figure 6B). Nevertheless, their relative contributions varied substantially in response to dietary glucose elevation and SchB intervention. Zebrafish fed an HGD displayed a significant expansion of *Aeromonas*, an opportunistic genus frequently associated with intestinal imbalance, accompanied by reduced proportions of *Cetobacterium* and *Fusobacterium*. In contrast, SchB supplementation was associated with a marked decrease in *Aeromonas* abundance and a concomitant increase in *Cetobacterium* and *Fusobacterium* levels (Figures 6H–L).

### 3.7 Beta diversity and potential pathogenicity analysis

**Figure 7:**
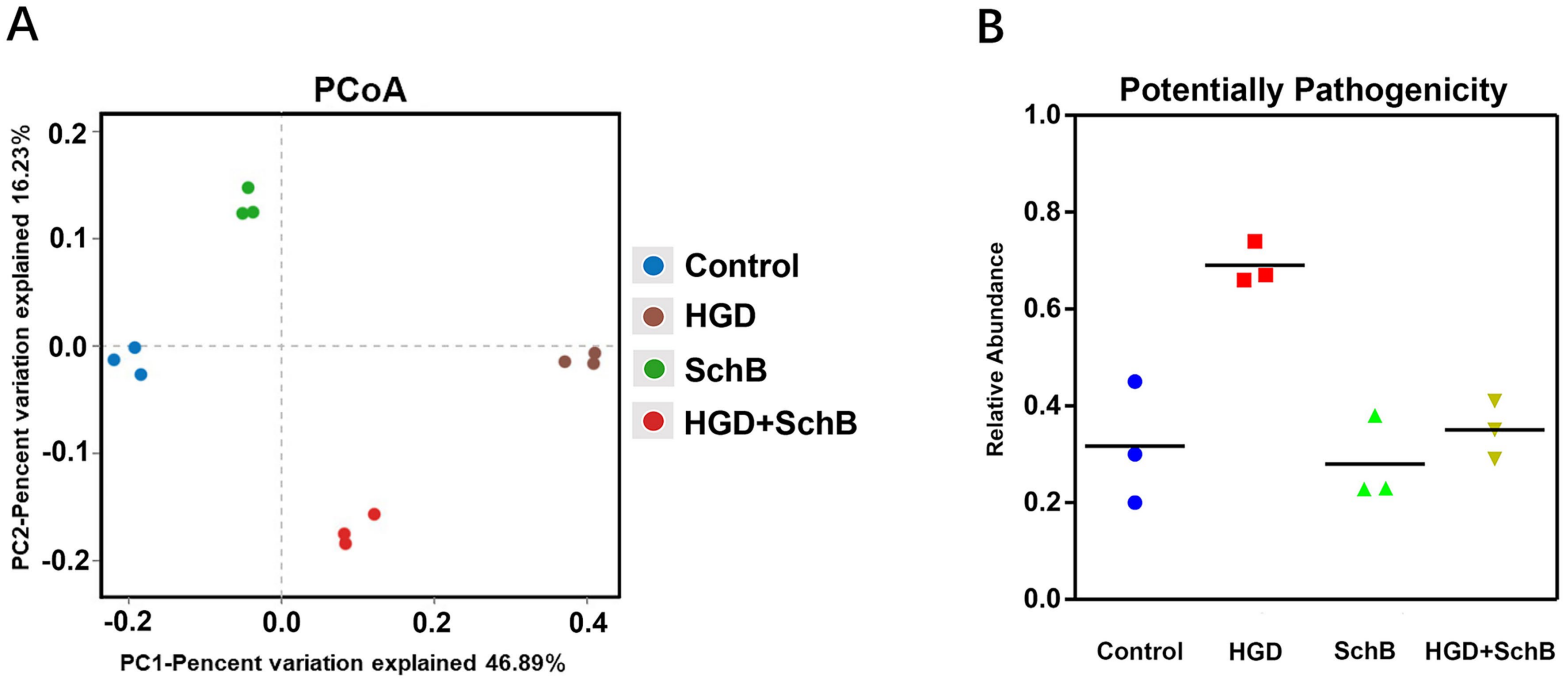
Beta diversity and predicted potential pathogenicity of the gut microbiota in different groups of zebrafish. **(A)** Principal coordinates analysis (PCoA) of the bacterial community at the genus level based on Bray–Curtis distances. Group differences were statistically evaluated using PERMANOVA. **(B)** Predicted potential pathogenic phenotypes of the gut microbiota inferred using BugBase.

PCoA was performed to assess beta diversity. Figure 7 illustrates the distinct clustering of intestinal microbiomes between the control group and treatment groups of fish. PCoA based on Bray–Curtis distances showed distinct clustering of microbial communities among groups. PERMANOVA analysis confirmed significant differences in community structure (*p* < 0.01). BugBase analysis revealed that SchB supplementation significantly reduced the relative abundance of potentially pathogenic bacteria and increased stress-tolerant phenotypes in the gut microbiota.

## 4 Discussion

Persistent elevation of blood glucose initiates a harmful sequence of events, with toxic reactive species playing a central role. This results in oxidative stress that harms a wide range of biomolecules, including lipids, proteins, and DNA, leading to a significant metabolic impairment consistent with previous reports (26). Glucotoxicity describes the detrimental effects of elevated blood glucose and excessive carbohydrate consumption on cells and tissues. High-carbohydrate diets enhance the expression of key lipogenic enzymes, thereby promoting liver fat synthesis and contributing to hepatic steatosis. Recent research also suggests that glucotoxicity can harm hepatocytes by triggering endoplasmic reticulum stress, ultimately resulting in hepatocyte dysfunction and cell death (27). Moreover, oxidative stress resulting from hyperglycemia exacerbates liver injury, as demonstrated in numerous studies using diabetic animal models (28).

The brain, an organ with high energy requirements, depends on efficient ATP generation via glycolysis, the tricarboxylic acid cycle, and oxidative phosphorylation. In hyperglycemic states, reducing sugars can react with lysine residues on proteins, resulting in the generation of advanced glycation end products (AGEs) (29). Backlogging evidence from human studies and experimental animal models demonstrates that both hyperglycemia and hypoglycemia can induce alterations in brain structure and impair neurocognitive function (30). Sustained elevations in blood glucose, as observed in both Type 1 and Type 2 diabetes mellitus, contribute to chronic complications affecting the nervous system, vascular endothelium, and kidneys. Oxidative stress is pivotal to the onset and progression of these diabetic complications by promoting cellular injury and dysfunction (31). Consistent with these findings, our study demonstrated that a high-glucose diet induced significant oxidative stress–related cellular injury in the liver, retina, and brain of zebrafish, further highlighting the harmful effects of glucotoxicity-induced oxidative stress across multiple organs. In this context, the HGD group served as a disease model, while the control group represented physiological baseline conditions. The robustness of this model was reflected by concordant changes in oxidative stress indices, inflammatory gene expression, histopathology, and gut microbiota profiles.

Hyperglycemia activates the polyol pathway, leading to the upregulation of aldose reductase and the accumulation of sorbitol. This cascade contributes to oxidative stress through enhanced NADPH consumption and increased production of ROS and RNS. Antioxidants prevent the oxidation of molecules crucial for cellular processes and shield cells from oxidative stress-induced damage. *In vitro* studies using cultured hepatocytes and *in vivo* rodent experiments have demonstrated that SchB possesses antihyperlipidemic, antioxidant, anti-endoplasmic reticulum stress, and anti-inflammatory properties (28). Additionally, accumulating evidence highlights the neuroprotective capacity of SchB, largely attributed to its ability to mitigate oxidative stress–mediated neuronal damage (32).

SchB has been shown to suppress the protein expression of IL-1β and TNF-*α* and to decrease MMP-2/9 phosphorylation in the ischemic hemispheres. As a result, it offers protection against cerebrum inflammation and prevents metalloproteinase degradation (33). In addition, SchB has demonstrated the ability to attenuate microglia-mediated inflammatory responses by suppressing the production of proinflammatory cytokines such as TNF-α, IL-6, and PGE2. Our study revealed significant alterations in inflammatory factor levels in SchB-treated groups. These results support our hypothesis that SchB exerts potent anti-inflammatory effects, which are associated with reduced expression of several key inflammatory mediators involved in HGD-induced inflammatory responses.

Furthermore, SchB has been shown to mitigate oxidative stress by reducing MDA levels and enhancing SOD activity, thereby preventing muscle injury induced by hind-limb ischemia/reperfusion (34). Notably, SchB also exhibits tissue-specific antioxidant effects, including in the brain (29). In our study, SchB treatment resulted in marked improvements in oxidative and inflammatory status, characterized by increased SOD levels and reduced ROS and MDA levels across multiple tissues. Nrf2 signaling is highly intricate because it functions at the core of a broad regulatory network. Its expression is strongly linked to the degree of oxidative stress within cells. Among its downstream pathways, the Nrf2/HO-1 axis represents a key antioxidant defense mechanism present in nearly all cell types (35). The HO system consists of three isoenzymes: HO-1, HO-2, and HO-3, all displaying identical catalytic activity (36). In this study, a high-glucose diet activated the levels of Nrf2 and HO-1, indicating that high glucose induced oxidative stress in cells. However, SchB was able to restore the elevated expression of Nrf2 and HO-1 caused by the high-glucose diet, suggesting that SchB may contribute to the maintenance of cellular redox balance, although the precise involvement of the Nrf2/HO-1 pathway requires further investigation.

The liver and intestine serve as vital organs for digestion and nutrient absorption in fish. In our study, HGD induced marked pathological alterations in both tissues, including hepatocellular injury, shortening of intestinal villi, and thinning of the intestinal wall. In contrast, SchB demonstrated clear protective effects on hepatocytes and intestinal morphology. Growing evidence suggests that the gut microbiota serves as an important determinant of obesity and associated metabolic disorders, including dyslipidemia, inflammation, and impaired glucose tolerance (12). Previous research has demonstrated that diets high in glucose (HGD) or fructose (HFD) decrease the richness and diversity of the gut microbiota, often leading to an elevated abundance of Proteobacteria and a reduced proportion of *Actinobacteria* in the intestines of mice (37). In this study, HGD markedly decreased the richness and diversity of the zebrafish gut microbiota. At the phylum level, HGD increased the relative abundance of *Proteobacteria* while reducing *Firmicutes*. At the genus level, it elevated opportunistic pathogens such as *Aeromonas* and decreased beneficial microbes like *Cetobacterium*, which may account for the increased potential pathogenicity observed in the HGD group. Conversely, SchB supplementation enhanced both the abundance and diversity of the intestinal microbiota, reduced the relative abundance of opportunistic bacteria, and promoted beneficial bacterial populations. These findings indicate that SchB supplementation is closely associated with marked improvements in gut microbiota composition, which coincide with reduced oxidative stress and inflammatory responses under high-glucose dietary conditions. Although clear alterations in microbial diversity and composition were observed, direct correlation analyses between specific bacterial taxa and host phenotypes were not performed in the present study. Therefore, the contribution of gut microbiota to the protective effects of SchB should be interpreted as a strong association rather than a confirmed causal relationship.

## 5 Conclusion

In summary, this study demonstrated that SchB alleviates high-glucose diet (HGD)–induced inflammation, oxidative stress, and liver and intestinal injury in zebrafish, and is accompanied by marked alterations in the composition and structural characteristics of the intestinal microbiota. SchB supplementation increased microbial richness and diversity, reduced the relative abundance of opportunistic bacteria, and enhanced the abundance of beneficial bacterial taxa. Importantly, improvements in oxidative stress and inflammatory responses were closely associated with concurrent changes in gut microbiota structure. Collectively, these findings suggest that SchB exhibits protective potential against HGD-induced physiological dysfunction in zebrafish and provide a theoretical basis for its further exploration in nutritional and pharmaceutical applications.

## Data Availability

The original contributions presented in the study are publicly available. This data can be found here: The sequencing data have been deposited in the Genome Sequence Archive (GSA) of the China National Center for Bioinformation (CNCB-NGDC) under accession number PRJCA062488.
